# Radular Teeth Morphology in *Limax* (*Caspilimax) keyserlingi* (Martens, 1880) and *Parmacella ibera* (Eichwald, 1841) from Northern Iran

**Published:** 2013

**Authors:** M Yakhchali, T Gorgani-Firuzjaei, K Baghri

**Affiliations:** 1 Department of Pathobiology, Parasitology Division, Faculty of Veterinary Medicine, Nazlu campus, Urmia University, Urmia, Iran; 2 Veterinary Parasitology, Faculty of Veterinary Medicine, Urmia University, Iran; 3 Veterinary Medicine, Faculty of Veterinary Medicine, Urmia University, Iran

**Keywords:** Radula, *Parmacella ibera*, *Caspilimax keyserlingi*, *Strongyloid*, Larvae, Iran

## Abstract

**Background:**

Slugs have been known worldwide as important pests of agricultural and horticultural production. They also play a role as intermediate or definitive hosts of helminths parasite. In this purpose, current study was carried out to examine slug radular teeth structure and slug infection with helminths larvae in north of Iran.

**Methods:**

A total number of 114 slugs were collected from center and east parts of Mazandaran province from May 2011 to June 2012. The specimens were rinsed, measured, and identified. The radula of all collected slugs was extracted and stained by using Mallory II. For detecting the helminths parasite infection, foot- head and viscera of examined slugs were removed, minced, and digested with 4.5% acid pepsin.

**Results:**

Two species of *Limax* (*Caspilimax) keyserlingi* (Martens 1880) (11.4%, 13/114) and *Parmacella ibera* (Eichwald 1841) (88.6%, 101/114) were prevalent in the region. There was significant difference between body length and shell size. *P. ibera* had the highest number of teeth rows (145±5). The radular teeth formula was approximately similar in both identified slugs. In *P. ibera*, there was no significant difference in the average length and width of radula. The radular teeth in *L. keyserlingi* were larger and thicker than *P. ibera*. In all examined slugs for helminths larvae infection, *P. ibera* (7.69%, 1/13) was infected with *Strongyloid* larvae from Fereidonkenar area.

**Conclusion:**

Two prevalent species of slugs exist in the same region of which *P. ibera* has capability to play a role as intermediate host of nematode helminths. Radular morphology within the slug species may be also systemically informative.

## Introduction

The molluscs are an old age group found among early fossils, a group of great diversity in size, distribution, habitat, and utility throughout the world. The range of their distribution is as extensive as it covers terrestrial, marine, and freshwater habitats (1). Slugs have been regarded worldwide as severe pests of agricultural and horticultural production, attacking a vast array of crops and plants (2). Recent studies indicated that these mulluscs are important because of economic losses in garden crops and paddy fields especially in north of Iran (3). Several terrestrial and aquatic molluscs may act as intermediate hosts of parasitic helminths and also implicate in transmission of many plant pathogens (4).

In 25,000 described nematode species, 3,500 species are parasitic nematodes of invertebrates (5). The orders of Oxyurida, Rhabditida, and Strongylida (eight families) spend their life cycle inside slugs (6), i.e. *Angiostrongylus cantonensis*, *A. costaricensis*, *Alloionema appendiculatum*, *Mermis nigrescens*, *Alloionema appendiculata*, and Metastrongyloidea (6, 7). They can also play a role as definitive host e.g. *Agfa flexilis* and *Hugotdiplogaster neozelandia* (6, 8).

The morphology and morphometry of radular teeth has been considered as one of the most commonly used sources of information for studying molluscan systematic. The radular teeth studies of molluscan are typically unique to a genus or specie which will strengthen the accuracy of species identification (9). The radula in slugs has a number of minute teeth arranged in transverse rows with one central tooth (C), many lateral teeth (L), and numbers of marginal teeth (M) (10). There was no information about common slug radula structure in Iran and slugs infection with parasitic helminths.

In this purpose, the present study was aimed to examine slug radula structure and infection with helminths larvae in north of Iran for the first time.

## Materials and Methods

### Study area

**Fig. 1:**
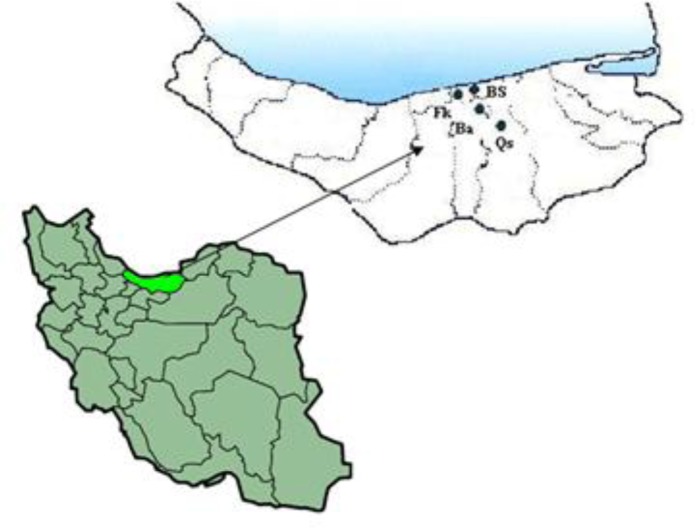
Map of North Iran showing the municipalities with samples of *Parmacella ibera* and *Limax keyserlingi* examined for nematode larvae (Ba, Babol; Bs, Babolsar; Fk, Fereidonkenar; Qs, Qaem Shahr)

Mazandaran province is located in north of Iran (50° 34′E, 36° 47′ N) with average rainfall of 700 mm, temperature 17°C, and humidity 79-80%. The province has four distinct seasons: cold season (January to March), spring (March to June), summer (July to September), and fall (October to December). The area of study is semi temperate with wet climate that divided into four sub-areas, i.e. Fereidonkenar, Babolsar, Babol, and Qaem Shahr (Fig. 1).

### Collection and identification of slugs

Over a period of one year from May 2011 to June 2012, a total number of 114 slugs were collected from old pieces of wood and leaves, stones, and flower pots of the study areas and transferred to Malacology Laboratory of Urmia Faculty of Veterinary Medicine. Before dissection on the right-hand side of the specimen, the length and width of the body slugs were measured and morphologic features were also recorded. The specimens were placed into a jar filled with boiled water that had been cooled and preserved in 70% ethanol until radula examination. Slugs were also taxonomically identified according to keys provided by Mansourian (10) and McDonnel et al. (11) with a magnifying glass.

### Preparation and examination of radular teeth

The slugs were examined using optic microscopy and then photographed. According to Yakhchali and Jamshidi Deilami (12), whole slugs were placed in a 90°C water bath for 5 minutes and then buccal mass including radula was completely removed. The radula was put into the 8% KOH for 24 h at 37°C and stained by Mallory II. Then, the cleaned radula was mounted with a mounting medium (Canada Balsam). The radular tooth located in central (C), lateral (L), and marginal (M) parts were measured using an Zeiss Standard compound microscope with an ocular micrometer of each tooth at ×400 and ×1000. To determine the size of radular teeth, the length of each tooth from the apical cusp to the base was considered. To find out teeth formula, the number of transverse rows and teeth in each row were also counted and recorded (12).

### Helminths larvae infection

To identify larvae infection, foot-head and viscera of the specimens were removed. The foot-head were individually minced and digested with 4.5% acid pepsin for 3h at 37°C. The digested samples were subjected to the centrifugation (2000 rpm per 5min). The viscera were also placed in Petri dishes with 0.01M phosphate-buffered saline (PBS, pH 7.2) solution. The viscera and sediment which collected for centrifugation were carefully examined for helminths larvae by using stereoscope. The collected larvae were washed in PBS and fixed in AFA and slides were prepared using lactophenol solution (12). The slides were examined under light microscope at ×400 magnification and identified on the basis of morphological and morphometric parameters (13, 14).

### Statistical analysis

Two sample student *t* test (SPSS version 16.0 for windows) were used to compare the differences of the length and width of radula teeth, slug body, slug shell among the seasons.

## Results

### Slugs’ diversity and abundance

In all examined slugs, two species of slug families, were identified i.e. Limacidea (Culvier, 1804) and Parmacellidae (Table 1). During the course of the study, the predominant slug was *Parmacella ibera* (Eichwald, 1841) (88.6%, 101/114) (Family: Parmacellidea, Lamarck 1801) which were found in all places of study. However, *Limax keyserlingi* (synonym, *Caspilimax keyserlingi*, Martens, 1880) (11.4%, 13/114) (Family: Limacidea) was found only in Fereidonkenar in spring 2011 (Table 1).

**Table 1:** Mean length and width of radula teeth, body, and shell size of *Limax keyserlingi* and *Parmacella ibera* (n = 114, Mean± SD)

Slug	Time	No. of examined slugs		Teeth (µm)				Slug (mm)		Slug shell (mm)	
				L	W	TR	TF	L	W	L	W
*Limax keyserlinigi* (Fk,52° 53′E, 36°39N, 13m ASL)			C	120±5.5	51.2±3						
	Spring (2011)	13	L	124±4	64±1.5	145±5	1-40-18	75±0	19.5±0.7	9±1	4±0.5
			M	127.03±5	35±6						
*Parmacella ibera* (Qs, 52° 53′E, 36° 28′N, 51.2m ASL)			C	115.9±12	60.7±4.7						
	Summer (2011)	12	L	116.6±6.5	56.5±1.5	115±5	1-40-15	70±2	9±1	10.1±1	5±0.5
			M	120.5±5.5	32.7±7						
*Parmacella ibera* (Bs, 52°64′ E, 36°71′N, 7m ASL)			C	109±11	61.5±4						
	Fall (2011)	35	L	115.5±9.5	59.8±4.5	115±5	1-40-15	35±4.5	5.5±0.5	7.5±0.3	3.5±0.4
			M	121.5±10.5	29±5						
*Parmacella ibera* (Qs, 52° 53′E, 36° 28′N, 51.2m ASL)			C	121±10	64±3						
	Winter (2011)	28	L	125.5±15	60.7±6.5	115±5	1-40-15	48±3	10.8±1	10.8±1	5.2±0.5
			M	126±10	38.6±7						
*Parmacella ibera* (Ba, 52° 41′E, 36° 33′N, 2m ASL)			C	118.5±18.5	55.2±1.5						
	Winter (2011)	26	L	119.35±6.5	55.2±1.5	115±5	1-40-15	66±1.7	13.5±5	10.3±0.7	5.2±0.3
			M	123.7±11	30.5±6.5						

Notes: ASL, above sea level; Ba, Babol; Bs, Babolsar; Fk, Fereidonkenar; Qs, Qaem Shahr; L, length; TF, teeth formula; TR, teeth rows; W, width;

### Slugs’ morphology

The morphology features of examined slugs were individual and different. The color of examined *P. ibera* was brown to gray. The slugs of Qaem Shahr were yellowish to brown. In addition, they became darker and prone to grey during the course of the study. The mantle covered a small part of the body. While in *L. keyserlingi*, body color was yellowish brown to grey and mantle covered most parts of the body. In *P. ibera*, the respiratory pore was smaller and posterior end of the body was blunt with dark gray spots. Two pairs of retractile tentacles (1-5mm) with two eyes at the end of long posterior tentacles were observed in both identified slugs. The sole color for *P. ibera* was yellow, while it was colorless for *L. keyserlingi*.

### Description of radular teeth structures

**Fig. 2:**
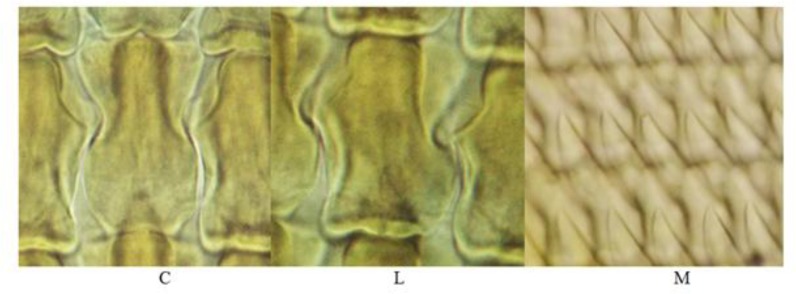
The radular central (C), lateral (L), and marginal teeth of *Limax keyserlinigi* (1000×, Mallory II staining)

**Fig. 3:**
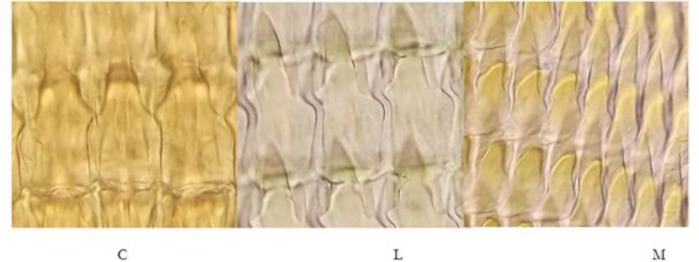
The radular central (C), lateral (L), and marginal teeth (M) of *Parmacella ibera* (1000×, Mallory II staining)

Figures 2 and 3 were shown various radular teeth in both identified slugs. The radular pattern appeared in all studied specimens. However, some morphological differences were observed in the specimens. The radular formula was approximately similar to *Parmacella ibera* (1C + 40L + 18M) and *L. keyserlingi* (1C + 40L + 15M) which *P. ibera* had the highest numbers of teeth rows (145±5) among examined slugs (P < 0.05, df = 18) (Table 1). The C tooth in the middle of each row of radula had specific cusp shape for *L. keyserlingi* in comparing with *P. ibera* (Figs. 2 and 3).

The L teeth were quadrangular in *L. keyserlingi* and triangular in *P. ibera* which was different in sizes (*L. keyserlingi*: 120±5.5 and *P. ibera*: 121±10) (P > 0.05). In *P. ibera*, the cusp of L teeth was sharp-pointed, while it was flat in *L. keyserlingi*. In *P. ibera*, there was no significant difference in the average length (5400±700) and width (1900±500) of radula (df = 35, *P*>0.05). The M teeth were sharp-pointed in *L. keyserlingi* and provided by a thin basal part.

The sharp-pointed cusp of M teeth in *P. ibera* was folded. The radular teeth (C, L, and M) were found larger (C: 115±14.5, L: 118.2±16.8, M: 122.7 ±10) and thicker (C: 62.9± 4.9, L: 57.55± 4.7, M: 32.8± 7.4) in *L. keyserlingi* than *P. ibera* (Figs. 1 and 2). Central tooth of *P. ibera* had a narrow cusp and two short and wide cusps. In lateral teeth, mesocone was larger and slit. Ectocones (22.5±3.7) were shorter in which near to marginal teeth (37.7 ±6.3).

### Slugs’ larvae infection

**Fig. 4:**
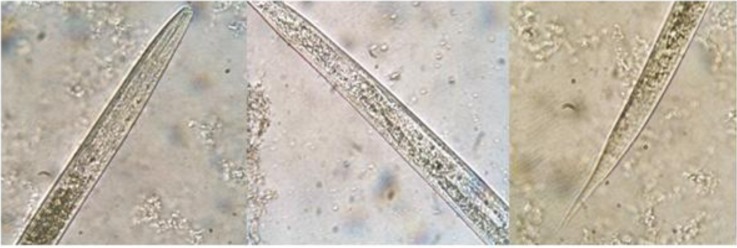
Strongyloid larvae removed from *Parmacella ibera* of Fereidounkenar (400×)

In all examined slugs for nematode larvae infection, *P. ibera* (7.69%, 1/13) was found to be infected with Strongyloid larvae with average length of body in 197±12.15µm and pointed tail with average length of 23±1.37µm from Fereidonkenar area in spring 2012 (Table 1) (Fig. 4).

## Discussion

Gastropod molluscs are adapted to marine, freshwater, and terrestrial life. They are very numerous and diverse in moist and humid environment in north of Iran. Slugs are present in traditional agricultural areas as well as, today, in landscaped urban and suburban areas (at least in this region). A number of terrestrial mollusc species have been introduced by extensive public, private landscaping, and agricultural activities using imported plants, soil, and fertilizer.

In this study, the most common slug species were *L. keyserlinigi* and *P. ibera*. *Limax* (Linnaeus, 1758) species may form daytime aggregations in daytime resting shelters, where they eventually may lay eggs (15). They were distributed in Europe, North Africa, and Asia Minor, with slugs commonest in the Mediterranean region (16). *L. keyserlingi* were distributed in SE Caucasus Mts., nearby Iran (17); Talysh and Lenkoran lowland forests (18). *Parmacella* (Cuvier, 1804) is a nocturnal slug which hides during daytime. This genus of slugs distributed over Canary Islands and Europe to Afghanistan (19). Ahmadi (3) and Mansoorian (10) reported both *L. keyselinigi* and *P. ibera* terrestrial slugs in Fereidonkenar in northern Iran. Lots of parasites and molluscs have been reported from northern Iran as a result of suitable environmental factors, i.e. humidity, rainfall, temperature, and human agricultural activities. Seasonal investigation indicated that the slugs’ population was the lowest in summer. It may be due to decreasing humidity and increasing temperature, since the slugs survive closely on the basis of environmental conditions (20).

The body features and structure of molluscan radular teeth are often unique for genus and species discrimination (12). In molluscan systematic, the radular features and measurements are traditionally informative to discriminate the slug genus or species (21). In this case, a detailed description of the radular teeth morphology and morphometry of *L. keyselinigi* and *P. ibera* were brought for the first in this work. In fall, newly hatched slugs were small and had more light color in spring than winter. Furthermore along with growing up the slugs, the radular teeth sizes, shell and body sizes increased. There was also significant difference between body length and shell size. These findings were in agreement with other studies (20, 22). The morphology of radular teeth (C, L, and M) in both species was similar. The average of length and width of C, L, and M teeth had significant difference in *P. ibera*. In a previous investigation on *Cryptella* (Gastropoda:Parmacellidae), similar features was reported by Huttere and Groh (23).

In current study, the Strongyloid larvae were found alive in body cavity of the infected slugs. The tip of the head of examined larvae was smooth and the esophagus was strongyle form and club shape (64.6±4.78µm). The intestinal cells appearance was not much clear, however, they were set in line of one row. According to Wyk et al. (14), the larvae of the helminths are generally easily identified on the basis of conventional characteristics. This is often based on distinguishing larvae features such as the shape of the head, tail and esophagus, intestine cell appearance, the length of the body and tail of the larvae. In contrast with parasitic nematodes larvae features, free-living larvae are relatively thick, cigar-shaped and with long tails, and have no covering sheaths as in the majority of the parasitic nematodes. The tip of the head is not smooth and the oesophagus is also markedly rhabditiform, with two conspicuous bulbs (24, 25).

With regards to the recorded larvae features, Strongyloid larvae infection was found in *P. ibera* with low frequency. It was previously reported that slugs may play a role as intermediate and or definitive host of helminths (6). Laznik et al. (26) noted the infection of Arionidae slugs with *Alloionema appendiculatum* (Family: Alloionematidae). In Iran, Karimi et al. (27) reported that *P. ibera* was infected with *Phasmarhabditis hermaphrodita*. The potential of the slugs as intermediate host for helminths is of great importance due to regional extensive livestock farming and human activities. In addition, they can serve as intermediate host for helminths infection in wild animals becoming as a risk factor for domestic animals of the region.

## Conclusion

Radular teeth morphology and morphometry within the slugs’ species could be one of the character sets for classification of slugs. Additionally, *P. ibera* infection to the nematode larvae elucidated further investigations need to study the role of other slugs for endemic helminths parasites, which are of medicine and veterinary importance in northern Iran.
